# Research on Eddy Current Probes for Sensitivity Improvement in Fatigue Crack Detection of Aluminum Materials

**DOI:** 10.3390/s25196100

**Published:** 2025-10-03

**Authors:** Qing Zhang, Jiahuan Zheng, Shengping Wu, Yanchang Wang, Lijuan Li, Haitao Wang

**Affiliations:** 1 College of Electrical Engineering and Control Science, Nanjing Tech University, Nanjing 211816, China; zhangqing@njtech.edu.cn (Q.Z.); 18652010963@163.com (J.Z.); ljli@njtech.edu.cn (L.L.); 2 Directly Affiliated Branch Institute, Special Equipment Safety Supervision Inspection Institute of Jiangsu Province, Nanjing 210009, China; wspcn@sina.com; 3 713th Research Institution, China State Shipbuilding Corporation Limited, Zhengzhou 450052, China; 13203734566@163.com; 4 College of Automation, Nanjing University of Aeronautics and Astronautics, Nanjing 211106, China

**Keywords:** fatigue cracks, eddy current probe, magnetic field focusing, directional selectivity

## Abstract

Aluminum alloys under long-term service or repetitive stress are prone to small fatigue cracks (FCs) with arbitrary orientations, necessitating eddy current probes with focused magnetic fields and directional selectivity for reliable detection. This study presents a flexible printed circuit board (FPCB) probe with a double-layer planar excitation coil and a double-layer differential receiving coil. The excitation coil employs a reverse-wound design to enhance magnetic field directionality and focusing, while the differential receiving coil improves sensitivity and suppresses common-mode noise. The probe is optimized by adjusting the excitation coil overlap and the excitation–receiving coil angles to maximize eddy current concentration and detection signals. Finite element simulations and experiments confirm the system’s effectiveness in detecting surface cracks of varying sizes and orientations. To further characterize these defects, two time-domain features are extracted: the peak-to-peak value (ΔP), reflecting amplitude variations associated with defect size and orientation, and the signal width (ΔW), primarily correlated with defect angle. However, substantial overlap in their value ranges for defects with different parameters means that these features alone cannot identify which specific parameter has changed, making prior defect classification using a Transformer-based approach necessary for accurate quantitative analysis. The proposed method demonstrates reliable performance and clear interpretability for defect evaluation in aluminum components.

## 1. Introduction

Aluminum alloys are commonly used in aerospace, transportation, and energy equipment owing to their light weight, corrosion resistance, and high electrical conductivity [1,2]. However, long-term operation or repetitive stress makes some areas of aluminum alloys, particularly joints, welded regions, and stressed components, susceptible to fatigue cracking (FC) [3,4]. FC grows from a small scale to a significant one, proliferating rapidly under increased stress, which may lead to material failure [5]. Thus, the detection of FC is essential for the integrity of aluminum alloy.

Eddy current testing (ECT) has been widely employed for aluminum alloy crack detection owing to its non-contact nature, rapidity, and sensitivity to surface defects [6,7,8]. While the orientation of the FC is uncertain, it is influenced by several factors, such as stress direction, crystal structure, and external stresses [9,10]. ECT has varied sensitivity when detecting cracks at different orientations [11,12]. It has poor sensitivity for detecting cracks that are not perpendicular to the induced eddy current caused by the probe. Moreover, early FC is narrower, requiring excellent precision of the ECT probe [13]. Therefore, it is essential to develop a probe that is highly sensitive to FC in any orientation.

Research has been carried out in the past few years, and some directional ECT probes have been developed for crack detection in any orientation. Wu et al. [14] designed a Tx-Rx coil sensor with an 8-shaped excitation coil and a circular receiver coil, demonstrating effective directionality for the detection of carbon-fiber-reinforced plastic (CFRP). Wang et al. [15] developed a probe that combines a right-angled triangular excitation coil and a TMR sensor array, which can accurately detect defects in all directions. Ge et al. [16,17] employed a rotating ECT probe that is excited by two sinusoidal waves of identical amplitude but with a 90° phase difference, to produce directional eddy currents for the identification of complicated cracks. Furthermore, some focused probes have also been proposed to enhance sensitivity. Xu et al. [18] developed a rotating focusing ECT probe by connecting four coils to form two figure-8-shaped focusing sub-probes, which are powered by two identical harmonic currents with a 90-degree phase difference. Trung et al. [19,20] developed a double semi-circular ECT probe that focuses eddy current on the surface of the specimen via a copper core. Liu et al. [21] proposed a semi-ellipsoidal eddy current probe with a magnetic flux-convergence design, incorporating a curved trapezoidal cross-section and a super-permalloy core. This configuration enhances magnetic field focusing and improves defect detection sensitivity through optimized structural parameters. Wang et al. [22] developed a Koch curve-based ECT probe that improves electromagnetic field focusing via spatial degree of freedom design, thus enhancing sensitivity to defects at different depths and orientations. However, the previously proposed probes are bulky and unsuitable for specimens with curvature. The ECT probe, constructed from flexible printed circuit board (FPCB), is then examined. Li et al. [23] introduced a probe employing an FPCB that generates directional eddy currents, enabling the detection of cracks in various orientations on complex geometric surfaces. She et al. [24] introduced a floral eddy current probe, in which the excitation coils are arranged in reverse order and symmetrically positioned. This configuration focuses the magnetic field and generates directional eddy currents through periodic distribution. Chen et al. [25] developed a fractal geometry-based FPCB probe that improves sensitivity to cracks in various orientations. To further improve its performance, they included a magnetic particle composite film into the probe, which focused the magnetic field and increased the eddy current intensity, thereby enabling more accurate detection of cracks in various orientations [26]. Trung et al. [27] designed a flexible eddy current (FEC) sensor with co-directional interleaved square coils. This compact structure enhances magnetic field focus and spatial resolution, enabling effective small crack detection at low frequencies. Ma et al. [28] presented a flexible square winding array structure that employs a double-period square excitation coil to improve directivity and focusing effects, hence augmenting the ability to detect small cracks. Compared to bulky ECT probes, only a few FPCB probes simultaneously achieve effective magnetic field focusing and the capability to detect defects across multiple orientations.

This paper proposes a focusing and directional FPCB probe aimed at improving sensitivity for the detection of FC in any orientation. The probe consists of an excitation coil and a receiving coil. The excitation coil consists of a double layer with opposing winding orientations, producing a focused diamond-shaped eddy current distribution on the surface of the aluminum alloy. The receiver coil employs a differential dual-layer configuration that enhances signal variations caused by cracks. The rest of the paper is organized as follows: Section 2 presents the design and optimization of the probe. Section 3 analyzes the probe’s ability to detect defects at varying angles and compares the responses of the designed coil with the commonly used rectangular coil. Section 4 describes the setup of the experimental platform. Section 5 focuses on the analysis and evaluation of defects. Section 6 presents the conclusions.

## 2. Probe Design

### 2.1. Probe Structure

**Figure 1:**
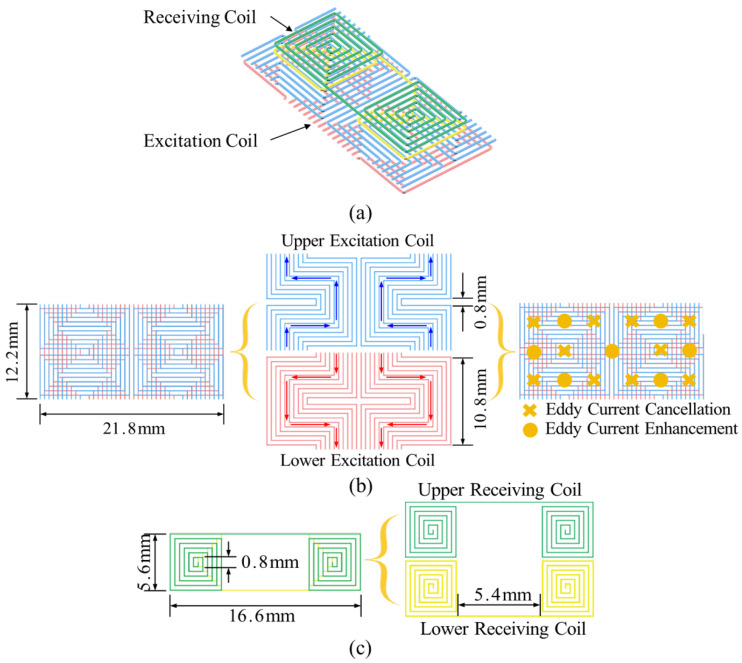
Proposed probe structure: (**a**) Integrated coil. (**b**) Excitation coil. (**c**) Receiving coil.

The proposed probe structure is illustrated in Figure 1a, where the receiver coil is positioned above the excitation coil. The excitation coil, depicted in Figure 1b, features a double-layer construction, with the winding orientations of the upper and bottom coils being opposite. The current of the excitation coils is illustrated in Figure 1b, with blue and red arrows denoting the current directions in the upper and bottom coils, respectively. The configuration of the excitation coil is pivotal in influencing the eddy current field on the specimen’s surface. Regions with opposing current directions in the upper and bottom coils diminish eddy currents in the specimen, whereas regions with same directions enhance eddy currents. As shown in Figure 1b, “×” represents the region where eddy current is smaller, whereas “∘” indicates the eddy current focus region. The distribution of eddy currents on the specimen’s surface displays a diamond pattern. This not only focuses the eddy currents but also introduces a directional eddy current distribution in the specimen, which is beneficial for FC detection. Moreover, as shown in Figure 1c, two coils with the same structure but opposite winding directions are used to form the differential receiver coil, which is used to further increase the sensitivity for FC detection. The left and right halves of the probe are symmetrical to increase the detection area and improve detection efficiency.

### 2.2. Simulation Analysis

**Figure 2:**
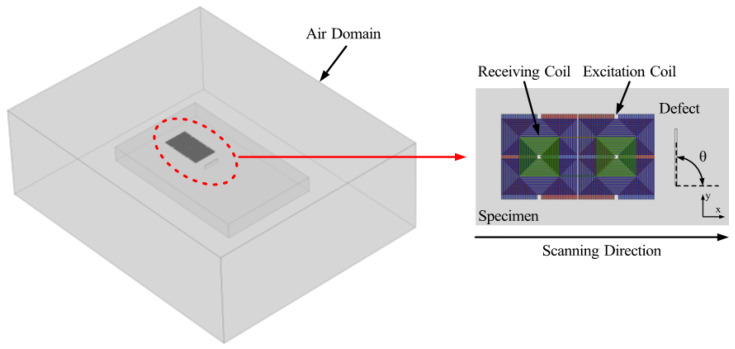
Finite element simulation model for defect detection analysis.

**Figure 3:**
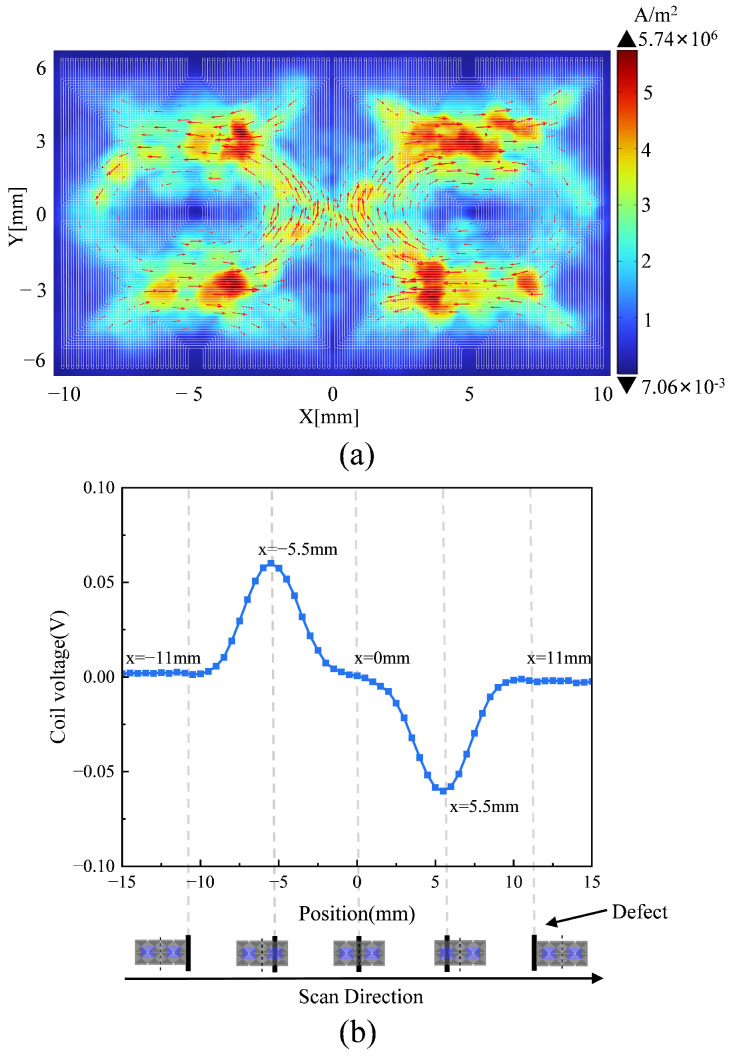
Simulation analysis of eddy current distribution and sensor response at 1 MHz: (**a**) Eddy current distribution on the test specimen. (**b**) Variation in sensor response when scanning for defects.

Simulation analysis is conducted to investigate the performance of the proposed probe. The AC/DC module of COMSOL Multiphysics 6.2 is employed in the simulation analysis, and the simulation model of the proposed probe is shown in Figure 2, in which the probe is situated 0.5 mm above an aluminum plate that has an FC with 8 mm length, 0.35 mm width, and 2 mm depth. Table 1 presents the other parameters of the simulation model, including the permeability, conductivity, size of the coil, the specimen, and the air around the specimen. A Cartesian coordinate is established for description, with the crack’s center position used as the origin. The crack’s angle is defined as the angle formed between the crack and the *x*-axis. The angle of the crack seen in Figure 2 is 90°. To simulate scanning, the displacement of the probe is realized through a parametric sweep along the *x*-axis. The coil position is updated step by step from *x* = –15 mm to *x* = 15 mm while maintaining a constant lift-off, and the results are depicted in Figure 3.

Figure 3a depicts the eddy current field on the specimen’s surface induced by the proposed excitation coil at 1 MHz. The distribution of eddy currents is focused on the specimen’s surface in a diamond pattern, which is consistent with the analysis presented in Section 2.1. Moreover, the signal for 90° FC is shown in Figure 3b, where the *x*-axis denotes the probe position and the *y*-axis represents amplitude. As shown in Figure 3b, the intervals *x* ∈ (−15, −11) and (11, 15) mm indicate that the FC is outside the probe’s detection range (which is determined by the sizes of the receiver coil), resulting in a signal amplitude of 0 V; the interval *x*∈ (−11, 0) mm signifies that the right region of the receiver coil is positioned over the FC, with the signal amplitude initially increasing and then decreasing; and at *x* = −5.5 mm, which is the center of the right receiving coil that coincides with the defect, the signal amplitude reaches its maximum value. A similar phenomenon occurs with the left receiver coil, however with inverted polarity of the amplitude, and when *x* = 0 mm, which is the receiver coil’s center is positioned above the FC, its signal amplitude registers as zero. In summary, the suggested differential receiving coil is exclusively responsive to the FC and its placement, thereby enhancing the signal-to-noise ratio (SNR) and facilitating the analysis of FC.

### 2.3. Probe Optimization

**Figure 4:**
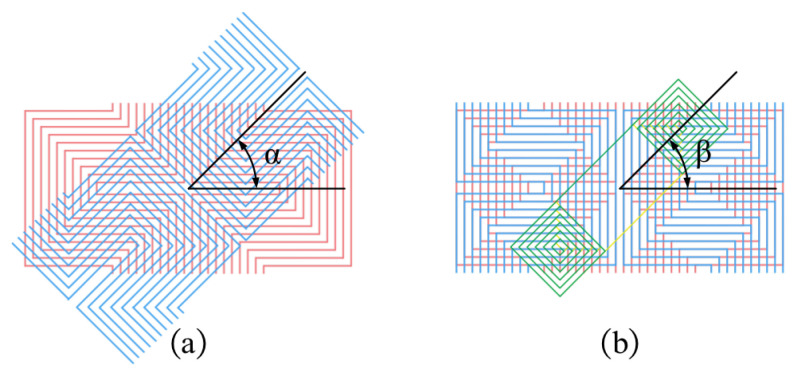
Schematic diagram illustrating the variation in coil angles: (**a**) Excitation coil overlap angle. (**b**) Receiving coil angle.

As shown in Section 2.2, the diamond-shaped eddy current distribution on the surface of the specimen is caused by the overlapping of the two excitation coils. Changing the angle of the two excitation coils will alter the eddy current distribution, which will impact the detected signals. Additionally, the detected signal may be impacted by the angle formed by the excitation and receiver coils, and optimization is performed in this section. As illustrated in Figure 4, the angle α is defined as the superposition angle between the upper and lower excitation coils, which governs the distribution of eddy currents on the specimen surface. The angle β is defined as the superposition angle between the excitation and receiver coils, which determines the sensitivity of the detection signals. Both α and β are 0° in the probe configuration depicted in Section 2.2.

**Figure 5:**
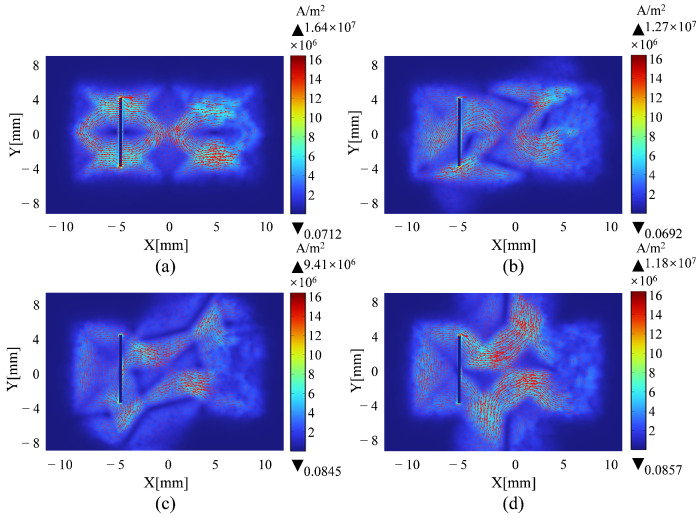
Eddy current density distribution with varying overlapping angles of upper and lower excitation coils: (**a**) 0°; (**b**) 30°; (**c**) 60°; (**d**) 90°.

**Figure 6:**
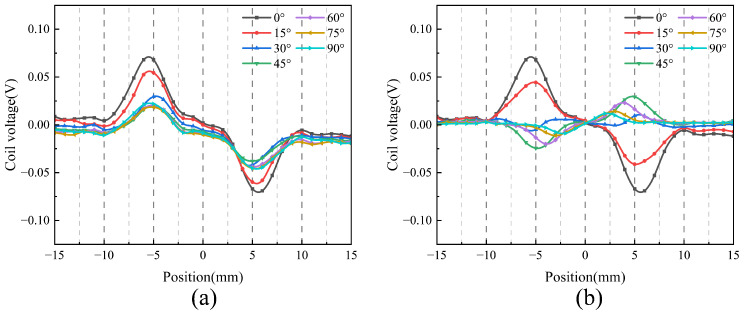
Relationship between coil voltage signal and coil angle variation: (**a**) Excitation coil overlap angle. (**b**) Receiving coil angle.

A simulation model similar to Figure 2 is used in this section. Firstly, the orientation angle α is optimized. Due to the structural symmetry, α is varied from 0° to 90° in 15° increments. The corresponding results are shown in Figure 5. As shown in Figure 5a, when α = 0°, the eddy current density reaches its peak, with a maximum value of $1.64\times 10^{7}\;A/m^{2}$; the induced eddy currents form a highly uniform and concentrated diamond-shaped pattern centered beneath the probe, indicating strong and effective excitation. As the angle α increases, both the intensity and the uniformity of the eddy current distribution gradually degrade. As shown in Figure 5b–d, compared with the 0° defect, all angled defects exhibit a reduced eddy current density and increased asymmetry in their distribution. Specifically, the maximum eddy current density decreases to $1.27\times 10^{7}A/m^{2}$ at 30°, $9.41\times 10^{6}A/m^{2}$ at 60°, and $1.18\times 10^{7}A/m^{2}$ at 90°. The eddy current progressively deviates from the central axis, and the overall field strength weakens significantly. At larger angles, the eddy current becomes more dispersed, indicating a decline in detection sensitivity. Furthermore, as illustrated in Figure 6a, the induced voltage in the receiving coil is highest at α = 0°, confirming that this orientation provides optimal excitation and detection performance.

Subsequently, the optimization of the receiver coil angle β is performed. β is varied from 0° to 90° in 15° increments. The results are shown in Figure 6b. The receiver coil voltage is maximized when β is set to 0°, which provides additional evidence that the best detection performance is achieved when both the excitation and receiver coil angles are set to 0°.

## 3. Simulation Analysis of FC with Different Angles

**Figure 7:**
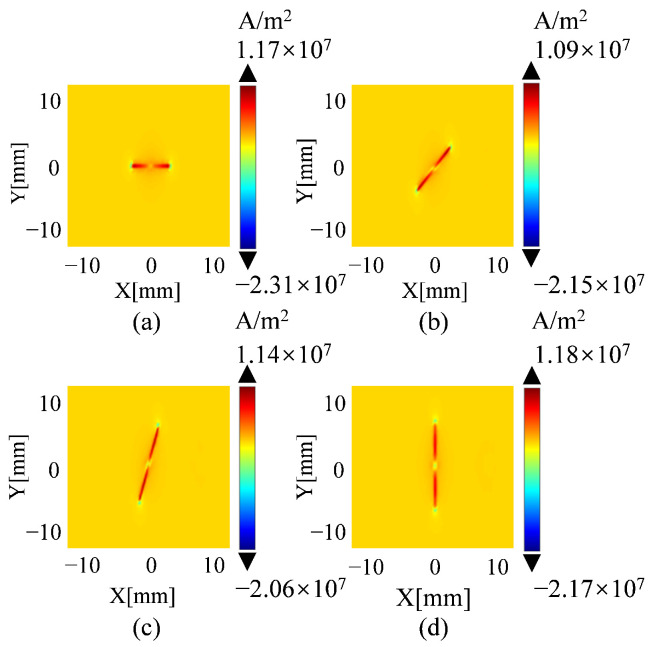
Eddy current density diagrams for the designed coil at different defect angles: (**a**) 0°; (**b**) 30°; (**c**) 60°; (**d**) 90°.

**Figure 8:**
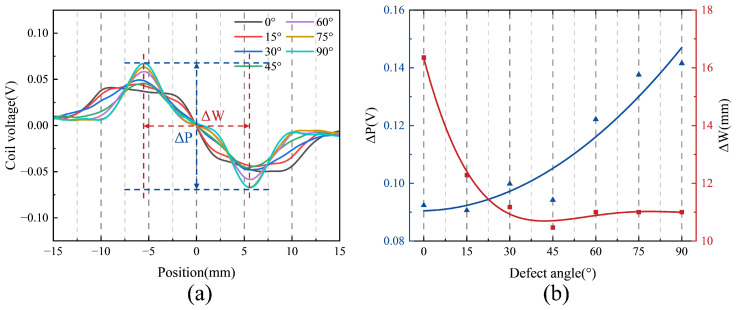
Coil voltage signals of the designed coil at different defect angles: (**a**) Comparison of test results. (**b**) Characteristic quantities.

To evaluate the probe’s capability in detecting defects with varying angles, simulations are conducted with defect angles set at 0°, 30°, 60°, and 90°, using a defect size 0.35 mm in width, 8 mm in length, and 2 mm in depth. The defect dimensions are consistent with those used in Section 2. Eddy current density is used for analysis, and it is defined as the difference between the eddy current distributions on the specimen surface with and without defects. Figure 7 presents the eddy current density with defect angles. Although the maximum eddy current density values are relatively consistent across all defect angles ($1.09\times 10^{7}$ to $1.18\times 10^{7}\;A/m^{2}$ ), the overall morphology of the eddy current distribution exhibits pronounced variations. The induced voltage signals, shown in Figure 8a, further confirm this trend. The highest response is observed at a defect angle of 90°, and the voltage amplitude gradually decreases as the angle decreases, highlighting the probe’s sensitivity variation with respect to defect orientation.

To further investigate the influence of defect angle on the detection signal, two time-domain features, ΔP and ΔW, are introduced [29]. ΔP denotes the peak-to-peak voltage, while ΔW represents the signal width, defined as the absolute difference between the *x*-coordinates corresponding to the peak-to-peak voltage, as illustrated by the red and blue markers in Figure 8a. Figure 8b presents the scatter plots and fitted curves of ΔP and ΔW as functions of the defect angle. As shown, both features exhibit clear dependencies on the defect angle, providing quantitative insight into how signal characteristics evolve with defect orientation. Specifically, ΔP increases progressively with defect angle, indicating greater signal variation due to changes in defect–probe interaction geometry. In contrast, ΔW decreases with increasing defect angle and gradually stabilizes, which can be attributed to a shorter interaction duration between the probe and the defect at larger angles.

**Figure 9:**
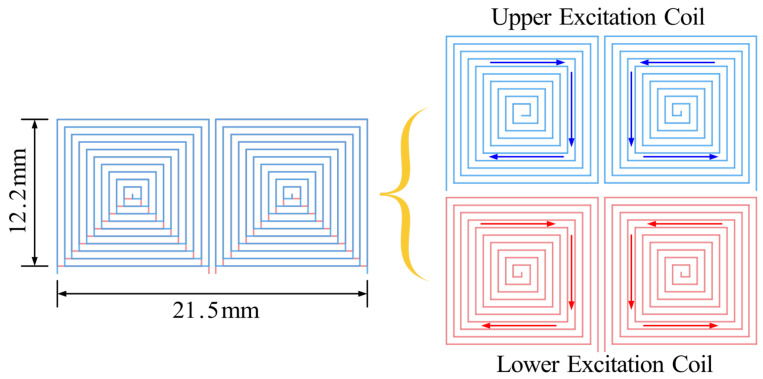
Rectangular coil structure design.

**Figure 10:**
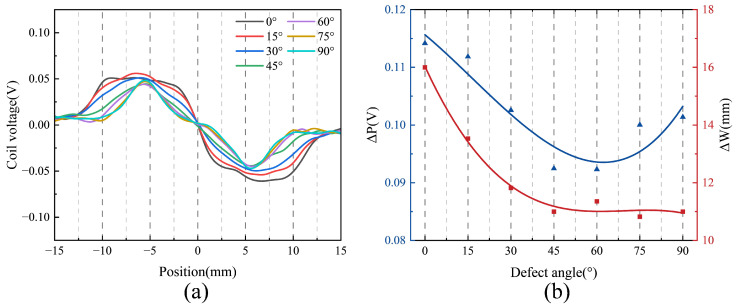
Coil voltage signals of the rectangular coil at different defect angles: (**a**) Comparison of test results. (**b**) Characteristic quantities.

To assess the effectiveness of the proposed flexible probe, its performance is compared with a commonly used rectangular coil under identical conditions. Figure 9 shows the rectangular coil structure, which has the same dimensions and wire diameter as the designed coil, and employs the same differential receiving coil to ensure consistent measurement conditions. Figure 10a presents the voltage signals of the designed coil at various defect angles, and Figure 10b displays those of the rectangular coil, including the scatter plot and fitted curve of ΔP and ΔW. The rectangular coil’s ΔP varies non-monotonically with defect angle, likely due to uneven eddy current distribution, which complicates accurate angle discrimination where signal amplitudes overlap. In contrast, the designed coil exhibits a monotonic ΔP increase, offering better resolution. Both show a decreasing ΔW with increasing angle. Overall, the proposed probe outperforms the rectangular coil in defect detection across angles due to its superior stability and discrimination.

## 4. Experimental Setup

**Figure 11:**
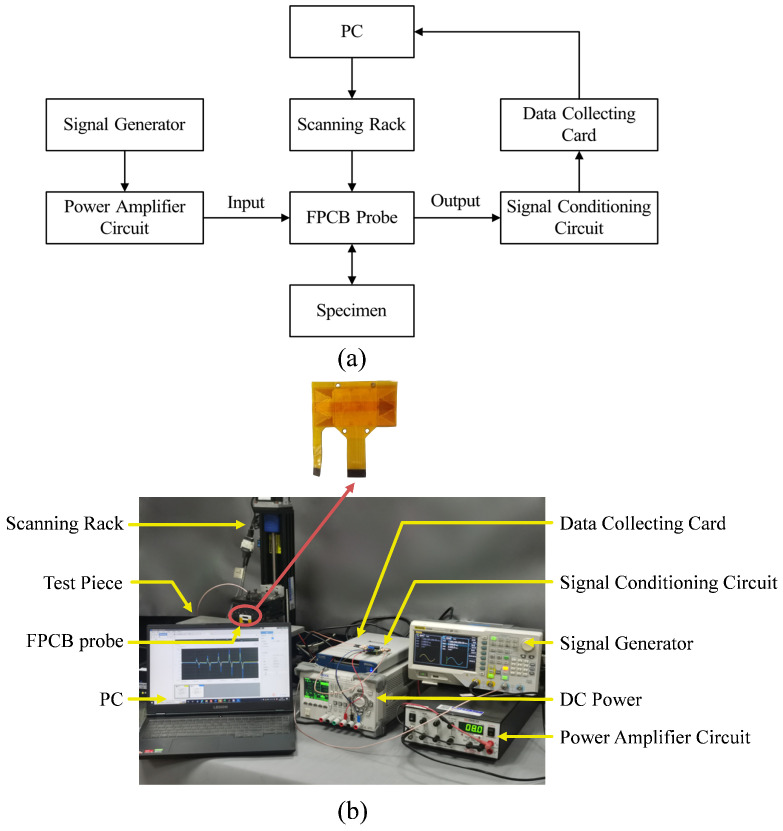
Experimental platform: (**a**) Schematic diagram of the experimental platform. (**b**) Physical drawing of the experimental platform.

The experimental setup is shown in Figure 11, where Figure 11a illustrates the experimental platform, and Figure 11b presents a physical representation. It comprises a signal generator, a power amplifier, a signal conditioning unit, a data collecting card, a scanning rack, a computer, an FPCB probe, and a flat panel specimen. The signal generator (DG4062, Rigol Technologies, Inc., Beijing, China) is used to generate two 1 MHz sine wave signals with an amplitude of 16 Vpp. One of the two generated signals is fed through the power amplifier to drive the excitation coil, providing a current of about 500 mA. The other signal, at the same frequency, is used as a reference for the signal conditioning unit. This reference signal is subsequently processed through a phase shifter to produce two orthogonal components, enabling precise quadrature demodulation. The power amplifier (Model 7602M, Krohn-Hite Corporation, Brockton, MA, USA) is used to amplify one of the signals and deliver it to the excitation coil of the FPCB probe to generate the primary magnetic field. The signal conditioning unit consists of a preamplifier (OPA1611, G = 11), a phase shifter (OPA1652, G = 1), and a quadrature demodulation module (AD630 + NE5532, G = 2). It is used to generate orthogonal reference signals from the second output of the signal generator and to process the alternating signal induced in the receiving coil. The total system gain is approximately 22, enabling effective amplification of weak sensor signals while maintaining a high signal-to-noise ratio. A fourth-order Butterworth low-pass filter is applied to suppress high-frequency components, reducing noise and improving overall signal quality. The data acquisition card (NI-6351, National Instruments Corporation, Austin, TX, USA) is used to synchronously collect the processed signals and transfer them to the computer via DAQExpress 5.0 software for storage and analysis. The scanning rack is used to hold and move the FPCB probe across the surface of the specimen during experiments, enabling automated scanning. As shown in Figure 11b, the FPCB probe consists of a double-layer excitation coil and a differential receiving coil. The detailed parameters of the probe are given in Table 2. At an excitation frequency of 1 MHz, the average resistance and inductance of the sensing units are 14.22 Ω and 4.28 μH, with fluctuations of about 0.5% and 0.7%, respectively.

**Figure 12:**
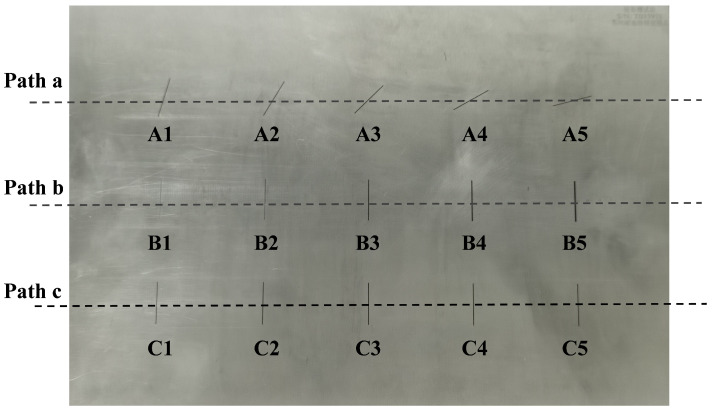
Aluminum sample with artificial defects.

In addition, the aluminum samples with defects are presented in Figure 12. Flat aluminum specimens with dimensions of 600 × 200 × 8 mm are used in the experiments. A series of artificial defects are introduced using Electrical Discharge Machining (EDM). These defects are uniformly spaced and increase progressively in size from left to right. To prevent interference between adjacent defects during testing, the probe scans the specimen along three predefined paths, denoted as paths a–c. Each path contains five defects, resulting in a total of fifteen defects. The detailed dimensions of the defects are listed in Table 3.

## 5. Discussion

### 5.1. Experimental Validation of ΔP and ΔW for Defect Geometry Characterization

To validate the simulation results presented in Section 3, experiments are conducted along the predefined scan path a using the experimental platform described in Section 4 to evaluate the probe’s response to defects with varying angles. Each defect is measured ten times under identical conditions, including a lift-off of 0.5 mm and a scanning speed of 1 mm/s. The sampling rate of the data acquisition card is 2000 samples/s. However, the raw signals from the data acquisition card are not used directly due to random errors caused by data acquisition and baseline drift induced by temperature. To address this, median filtering and detrending methods are applied, with the latter involving subtraction of an optimal least-squares fitting line to eliminate baseline drift. The repeated measurements are then used to obtain the mean and standard deviation of the characteristic parameters ΔP and ΔW, which serve to evaluate repeatability and experimental uncertainty.

**Figure 13:**
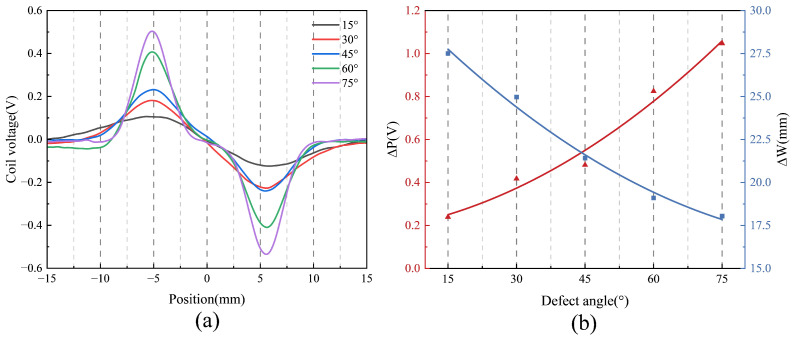
Defect detection results for defects of different angles: (**a**) Comparison of test results. (**b**) Characteristic quantities.

Figure 13 presents the experimental results for five defects with different angles. As shown in Figure 13a, the probe successfully detects all five defects, producing distinct signal responses characterized by variations in both amplitude and width, which are consistent with the simulation results. The responses also exhibit a high signal-to-noise ratio. Specifically, Figure 13b demonstrates that ΔP increases monotonically with increasing defect angle, aligning well with the simulation data presented in Figure 8. Moreover, ΔW decreases with increasing defect orientation, in general agreement with simulation results. Simulations indicate that ΔW tends to plateau at higher orientations, whereas experimental measurements show a continued decrease at the largest orientations, albeit with a diminished rate. Despite this minor deviation, the findings confirm that ΔW remains a robust feature sensitive to defect orientation.

To further assess the probe’s capability in detecting defects of varying sizes, experiments are conducted along the predefined scan paths b and c.

**Figure 14:**
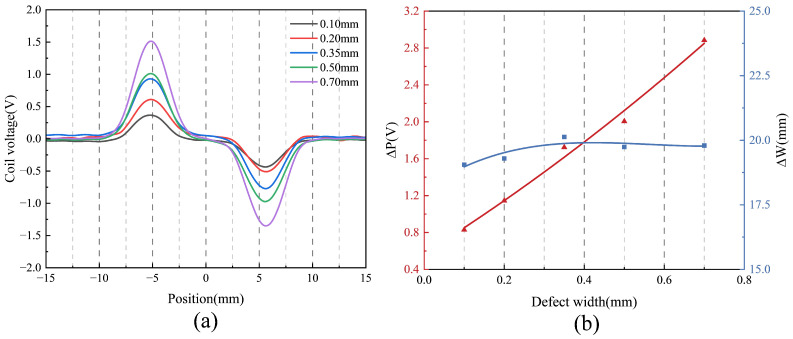
Defect detection results for defects of different widths: (**a**) Comparison of test results. (**b**) Characteristic quantities.

Figure 14 illustrates the probe’s response to defects of varying widths. As shown in Figure 14a, the black curves represent the response corresponding to the 0.1 mm defect, indicating that the probe reliably detects narrow cracks as small as 0.1 mm and demonstrates high spatial resolution. In Figure 14b, the parameter ΔP increases monotonically with defect width; this is because wider defects cause greater disruption to eddy currents, resulting in stronger signals. In contrast, ΔW remains approximately 20 mm with minor fluctuations, indicating low sensitivity to variations in defect width. This behavior contrasts with the angle experiments, where ΔW varied significantly. This is because variations in defect angle substantially change the orientation of the eddy current perturbation relative to the probe’s sensing field, thereby modifying the temporal evolution of the induced signal and producing pronounced changes in ΔW. In contrast, defect width mainly alters the lateral spread of the perturbation within the material, which exerts only a limited influence on the temporal width of the signal and thus results in minimal variation in ΔW.

**Figure 15:**
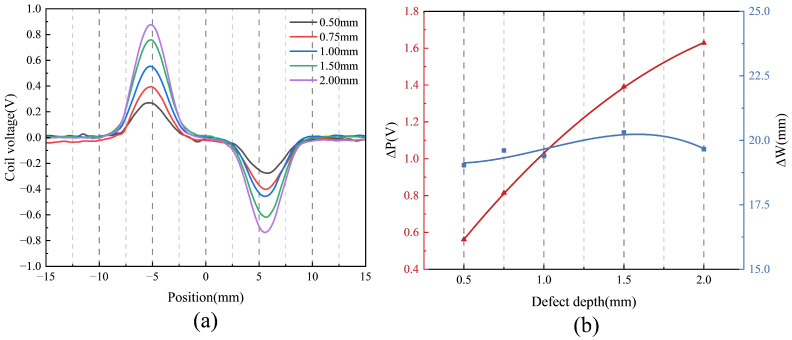
Defect detection results for cracks of different depths: (**a**) comparison of test results and (**b**) characteristic quantities.

Figure 15 presents the probe’s response to defects of varying depths. As shown in Figure 15a, the black curves represent the response corresponding to the 0.5 mm defect, demonstrating the probe’s ability to detect subsurface cracks as shallow as 0.5 mm with high sensitivity to vertical variations. In Figure 15b, as defect depth increases, the parameter ΔP rises proportionally, reflecting a greater disruption of the eddy current field. Conversely, ΔW remains approximately 20 mm with minor fluctuations, indicating low sensitivity to variations in defect depth. ΔW response to depth variations likely arises because depth primarily affects the intensity of the eddy current disturbance, with negligible impact on its spatial or temporal profile, resulting in a stable temporal width measurement.

The results demonstrate that ΔP is sensitive to variations in all defect parameters, including angle, width, and depth, exhibiting noticeable changes across these dimensions. Consequently, ΔP alone cannot definitively identify which specific defect dimension has changed. Similarly, ΔW primarily responds to changes in defect angle and exhibits limited sensitivity to width and depth. Notably, the variation ranges of ΔW corresponding to different defect parameters overlap significantly: 17.6 mm to 27.5 mm for angle, and approximately 20 mm for both width and depth. Due to this overlap, ΔW also fails to accurately distinguish the specific defect parameter, which causes difficulties in quantitative analysis. Therefore, it is necessary to classify defects prior to quantitative analysis.

### 5.2. Transformer-Based Autoencoder for Defect Feature Analysis

A Transformer-based autoencoder framework is introduced to classify defect types, thereby facilitating subsequent quantitative analysis. The Transformer is a neural network architecture based on self-attention, capable of capturing long-range dependencies and subtle temporal variations in sequential data, making it well suited for analyzing complex eddy current signals. Its parallel processing capability and ability to model non-local interactions contribute to its effectiveness in defect classification tasks.

Time-domain eddy current signals are collected from three defect types (angle, width, depth), with five representative signals per type, yielding 15 original signals. The dataset is expanded tenfold via data augmentation, including Gaussian noise injection, amplitude scaling, and time shifting. All signals are resampled to a uniform length and standardized, and auxiliary features—defect length, width, depth, angle, and two time-domain characteristics (ΔP, ΔW)—are also standardized. The signals are projected into a 32-dimensional embedding space and combined with auxiliary features to form the model input.

This combined input is processed through a single-layer Transformer encoder with four self-attention heads and a feedforward dimension of 128, ultimately producing a 128-dimensional latent representation for defect classification. The model is trained with five-fold cross-validation over 150 epochs using the Adam optimizer (learning rate $1\times 10^{-4}$, weight decay $1\times 10^{-4}$), with a composite loss function (0.7 × MSE + 0.3 × cross-entropy). Gradient accumulation over four steps is used to reduce memory consumption. The latent vector is then mapped through fully connected layers to generate final defect class probabilities, and the model with the lowest validation loss across all folds is selected for evaluation.

**Figure 16:**
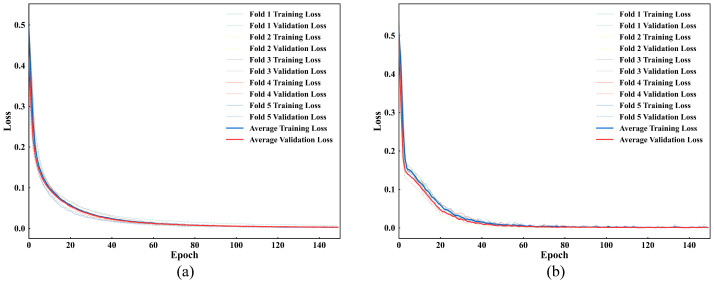
Training and validation loss curves: (**a**) Transformer and (**b**) CNN.

To establish a performance baseline, a CNN-based model is implemented with comparable model complexity and identical training settings as the Transformer. The CNN consists of three convolutional blocks with channels of 32, 64, and 128, using kernel sizes of 7, 5, and 3, each followed by ReLU activation and max-pooling. After adaptive average pooling to size 32, the features are flattened and concatenated with separately processed auxiliary features (6-dimensional input mapped to 64 dimensions), then projected to a 128-dimensional latent space matching the Transformer’s feature dimensionality. Both models are evaluated using five-fold cross-validation with identical data splits. The training and validation loss curves are shown in Figure 16. Both models show a steady decline in loss that levels off toward the end of training, indicating smooth convergence and stable training. The performance metrics for both models are listed in Table 4. The results indicate that the Transformer achieves slightly higher accuracy and precision compared with the CNN, demonstrating a consistent performance improvement across all metrics. Although the Transformer requires a somewhat longer training time due to its computational complexity, this additional cost is justified by the improved classification accuracy and robustness under varying inspection conditions. Furthermore, the Transformer framework can readily integrate auxiliary features such as frequency-domain characteristics or imaging data, which will be valuable for more complex defect characterization in future work.

**Figure 17:**
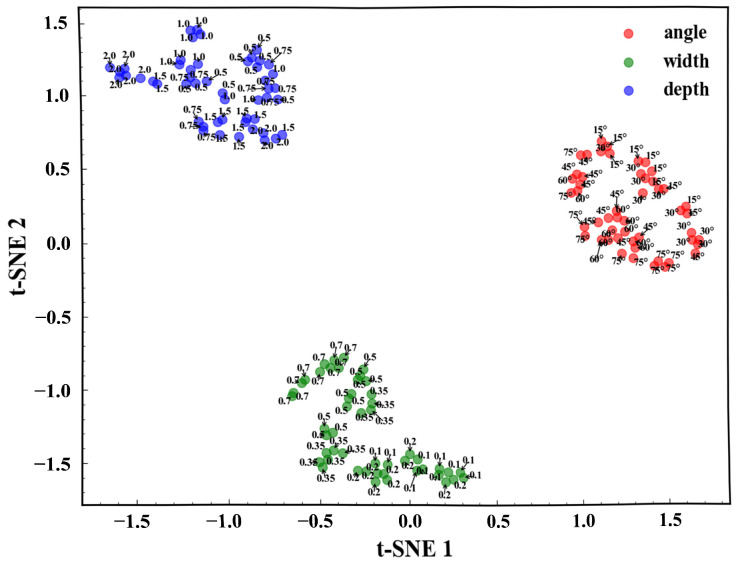
t-SNE visualization of the clustered latent features for different defect types.

To visually assess how well the Transformer autoencoder captures differences between defect types, the 128-dimensional latent features are first reduced to 50 dimensions using Principal Component Analysis (PCA) and subsequently clustered with KMeans, with the optimal number of clusters determined by the silhouette score. The features are then projected into two dimensions using t-distributed Stochastic Neighbor Embedding (t-SNE), resulting in well-separated clusters that correspond closely to the different defect types. Figure 17 shows the clustering results of different defect types. The visualized clusters exhibit clear separation and compactness, corresponding well with defect categories such as angle, width, and depth. This indicates that the proposed feature extraction and dimensionality reduction approach effectively captures the distinguishing characteristics of various defect types.

For practical defect detection, defect type identification should be performed as the preliminary step prior to quantitative analysis, since determining the defect category in advance allows the model to apply defect-specific regression strategies, thereby improving both the accuracy and efficiency of subsequent dimensional characterization.

## 6. Conclusions

The proposed FPCB probe demonstrates enhanced eddy current inspection performance through its double-layer planar excitation and differential receiving coil design. The excitation coil employs a reverse-wound configuration to enhance both the directionality and focusing of the magnetic field, while the differential receiving coil improves sensitivity and suppresses common-mode noise. Optimization of both the excitation and receiving coil angles maximizes eddy current density and detection signal strength. Moreover, finite element simulations and experimental validations show that the probe achieves high spatial resolution and robust sensitivity across a variety of surface cracks, while analysis of the time-domain features, ΔP and ΔW, shows that although they are sensitive to various defect parameters, overlapping responses necessitate a Transformer-based classification strategy for accurate quantitative evaluation. The proposed method demonstrates reliable performance and clear interpretability for defect evaluation in aluminum components.

## Figures and Tables

**Table 1:** Simulation parameters.

Parameter	Specimen	Excitation Coil	Receiving Coil	Air Domain
Materials	6061-T6	Copper	Copper	Air
Dimension (mm)	80.0/50.0/8.0	19.0/12.0/0.017	5.6/16.6/0.017	120.0/90.0/90.0
Relative permeability	1	1	1	1
Conductivity (S/m)	$2.87\times 10^{7}$	$5.99\times 10^{7}$	$5.99\times 10^{7}$	1

**Table 2:** Geometric parameters of the FPCB probe.

Coil	Wire Diameter (mm)	Wire Spacing (mm)	Wire Turns	Unit Width (mm)	Lateral Width (mm)
Excitation coil	0.1	0.1	26	\	47.85
Receiving coil	0.1	0.1	13	5.6	23.6

**Table 3:** Geometric parameters of defects.

Defect Number	Length (mm)	Width (mm)	Depth (mm)	Angle (°)
A1				75
A2				60
A3	20	0.35	2	45
A4				30
A5				15
B1		0.10		
B2		0.20		
B3	20	0.35	2	90
B4		0.50		
B5		0.70		
C1			0.50	
C2			0.75	
C3	20	0.35	1.00	90
C4			1.50	
C5			2.00	

**Table 4:** Performance comparison of the Transformer and CNN models.

Model	Epoch	Accuracy (%)	Precision (%)	Training Time (s)
Transformer	150	98.0	98.2	55.5
CNN	150	97.3	97.7	37.6

## Data Availability

Dataset available on request from the authors.
